# Factors Associated with Mortality in COVID-19 Patients: A Systematic Review and Meta-Analysis

**DOI:** 10.18502/ijph.v49i7.3574

**Published:** 2020-07

**Authors:** Mojtaba SEPANDI, Maryam TAGHDIR, Yousef ALIMOHAMADI, Sima AFRASHTEH, Hadiseh HOSAMIRUDSARI

**Affiliations:** 1. Health Research Center, Life Style Institute, Baqiyatallah University of Medical Sciences, Tehran, Iran; 2. Department of Epidemiology & Biostatistics, School of Health, Baqiyatallah University of Medical Sciences, Tehran, Iran; 3. Pars Advanced and Minimally Invasive Medical Manners Research Center, Pars Hospital, Iran University of Medical Sciences, Tehran, Iran; 4. Department of Epidemiology & Biostatistics, School of Public Health, Tehran University of Medical Sciences, Tehran, Iran; 5. Department of Public Health, Faculty of Health, Bushehr University of Medical Sciences, Bushehr, Iran; 6. Student Research Committee, Shiraz University of Medical Sciences, Shiraz, Iran; 7. Department of Infectious Diseases, Baharloo Hospital, Tehran University of Medical Sciences, Tehran, Iran

**Keywords:** Coronavirus disease 2019, SARS-CoV-2, COVID-19, Mortality, Comorbidities

## Abstract

**Background:**

The current study aimed to identify effective factors on the death among COVID-19 patients.

**Methods:**

All articles published in the period Jan 1, 2020, to Mar 23, 2020, written in English and reporting factors associated with COVID-19 mortality were reviewed. The random-effects model with 95% CI was used to calculate the pooled Odds Ratio (OR) and Hazard Ratio (HR). Data were analyzed using Stata ver.11.0.

**Results:**

The older age OR: 1.21(1.10–1.33) and male gender OR: 1.41(1.04–1.89) were most prone to death due to COVID-19. The Comorbidity with some chronic diseases such as Diabetes type2 OR: 2.42(1.06–5.52), Hypertension OR: 2.54(1.21–5.32), Kidney disorder OR: 2.61(1.22–5.60), Respiratory disorder 3.09 (1.39–6.88) and Heart diseases OR: 4.37 (1.13–16.90) can increase the risk of COVID19 mortality.

**Conclusion:**

Infection with COVID-19 is associated with substantial mortality mainly in older patients with comorbidities. We found the significant effect of age, gender and comorbidities such as Diabetes Mellitus, Hypertension, Kidney disorders and Heart diseases on the risk of death in patients with COVID-19. The factors associated with mortality found in this research can help to recognize patients with COVID-19 who are at higher risk of a poor prognosis. Monitoring these factors can serve to give early warning for the appropriate interventions.

## Introduction

Coronavirus Disease or (COVID-19), is an infectious disease caused by a new coronavirus that can transmit from one infected person to an average of 3 other people in a population (1). This emerging disease epidemiologically is similar to Severe Acute Respiratory Syndrome (SARS) and the Middle East Respiratory Syndrome (MERS), which were previously epidemic (2).

Although most of the people have mild symptoms, some may develop respiratory failure, arrhythmia, shock, renal failure, cardiovascular injury, or hepatic failure sometimes death (3, 4). At present, there is no effective antiviral treatment, only supportive care may be useful; such as mechanical ventilation, extracorporeal membrane oxygenation (ECMO) to patients with refractory hypoxemia, or ECMO to patients with refractory hypoxemia (4).

The overall Case Fatality Rate (CFR) is estimated to be 3.8%. CFR in patients with cardiovascular disease, diabetes, hypertension, respiratory diseases and cancer are estimated to be 13.2%, 9.2%, 8.4%, 8.0% and 7.6%, respectively (5). Several studies focusing on factors affecting the mortality of COVID-19 have been published in medical journals. Summarizing and combining the results of these studies are essential to provide convincing, and reliable evidence for clinical decision-making. Systematic reviews usually performed on clinical trials, they have also been used to combine and synthesize observational studies when interventional studies are not feasible (6). This is the case for the factors affecting the mortality of COVID-19.

We investigated the possible risk factors of death in patients with COVID-19, and determined the features that may predict mortality.

## Materials and Methods

### Eligibility criteria

All articles published in the period Jan 1, 2020, to Mar 23, 2020, written in English and reporting factors associated with COVID-19 mortality were reviewed. Cohort, case-control, or cross-sectional studies were included. Review articles as well as articles that lacked original data or reported incomplete data were excluded.

### Information sources and search strategy

We conducted a systematic review using Medline/PubMed, Scopus, and Google scholar. The following search terms used: “risk factors”, “COVID-19”, “coronavirus disease 2019”, “coronavirus disease-19”, “2019 novel corona-virus disease”, “severe acute respiratory syndrome coronavirus”, “mortality”, “death”. The searches were concluded by Mar 23, 2020, and two researchers independently assessed search results. References of related papers were also searched for other relevant articles to enhance the search strategy.

### Study selection

The results of the search strategy were initially screened based on abstracts and titles. The full text of related articles was then evaluated based on the inclusion and exclusion criteria. Observational studies that reported factors associated with death were included in the meta-analysis.

### Data collection process and data items

Data including the type and date of publication, country, the sample size, age, sex and other factors associated with COVID-19 mortality were extracted independently by two researchers. A third researcher checked the article list and data extractions to ensure there were no duplicate articles and resolved discrepancies about study inclusion.

### Assessment of methodological quality, risk of bias and publication bias

In order to quality assessment, we used the Newcastle Ottawa Scale for cohort, case-control and cross-sectional studies (7, 8). According to these checklists, the studies can be divided into three groups (good quality, Fair quality and poor quality). The publication bias was assessed using Begg’s and Egger’s tests.

### Meta-regression analysis

To assess the effect of sample size on pooled estimations the meta-regression analysis was used.

### Statistical approach

The random-effects model with 95% CI was used to calculate the pooled Odds Ratio (OR) and Hazard Ratio (HR), given variable degrees of data heterogeneity, and given the inherent heterogeneity in any systematic review of published papers from the literature. Considering the rarity assumption for death due to COVID-19 in a scenario we assumed OR and HR equal so we calculated pooled effect by combination OR and HR. The data were analyzed using Stata version 11.0. Pooled OR and its 95% confidence intervals (95% CIs) were used to summarize the weighted effect size for each study-grouping variable using the binary random-effects model. Measures of heterogeneity, including Cochran’s Q statistic, the I^2^ index, and the tau-squared test, were estimated and reported.

### Ethics Statement

This study was approved by the Ethical Committee of Baqiyatallah University of Medical Sciences with ID: IR.BMSU.REC.1399.095.

## Results

### Study selection and descriptive characteristics

**Fig.1:**
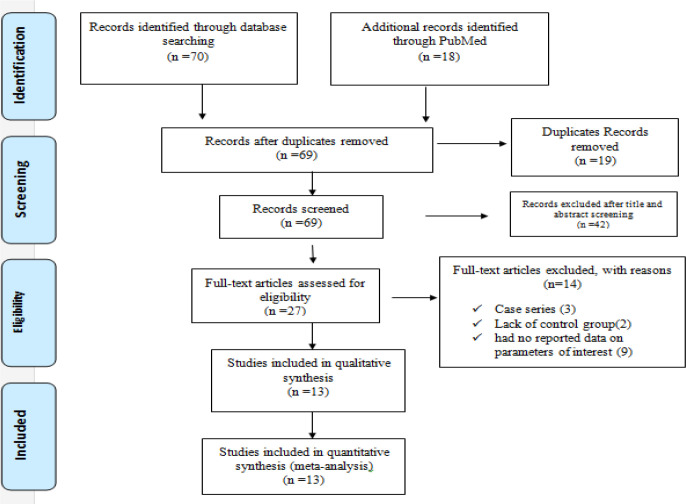
PRISMA Flow Diagram for included studies in current meta-analysis

In the current systematic review and meta-analysis, 13 studies that assessed the effective factors on COVID-19 mortality were included in final analysis. After searching PubMed and Google Scholar electronic databases, 88 possibly relevant articles were identified; 19 articles were removed due to duplication. Of the remaining 69 articles, 42 were excluded after screening based on abstract and title. Finally, 27 publications remained to be checked by reading their full text. Moreover, we excluded 14 articles after applying the inclusion and exclusion criteria: 3 articles were case series; 9 articles had no reported data on parameters of interest; 2 articles had no control group; in total, 13 eligible studies were included in meta-analysis (Fig. 1). All of included studies have been published during 2020 in China. The mean sample size was 944 (Range: 27 – 9651) (Table 1).

**Table 1:** Characteristics of the included studies on effective factors on mortality COVID-19, 2020

***First Author***	***Country***	***Year***	***Sample Size***	***Study type***	***Extracted Risk factor***	***Quality***
Jiatao Lu, et al (44)	China	2020	577	Retrospective one-center cohort study	Age, Sex, Hypertension, Diabetes mellitus	Good
Fei Zhou, et al (45)	China	2020	191	Retrospective cohort study	Age, Sex, Smoking, Chronic pulmonary disease, coronary heart disease, Diabetes mellitus, Hypertension	Good
Yichun Cheng, et al(46)	China	2020	701	Prospective cohort study	Age, Sex Acute kidney injury stage1 Acute kidney injury stage2 Acute kidney injury stage3	Good
Zhibing Lu, et al (47)	China	2020	123	Retrospective	Sex, Smoking, Hypertension Coronary heart disease Diabetes mellitus, Respiratory failure Malignancy, Chronic kidney disease Fever, Cough, Fatigue, Headache, Hemoptysis, Dyspnea	Good
ChaominWu, et al(12)	China	2020	201	Retrospective cohort study	Age, Sex, Fever, Hypertension Diabetes mellitus	Good
Yi Yang, et al (48)	China	2020	36	Retrospective cohort study	Age, Sex	Good
Qiao Shi, et al (29)	China	2020	101	Retrospective study	Age, Sex, Fever, Fatigue, Myalgia Headache, Hypertension, Dyspnea, Cough coronary heart disease, Diabetes mellitus Chronic pulmonary disease, Chronic kidney disease, Malignancy, Respiratory failure Acute kidney injury	Good
Xiaowei Deng, et al(49)	China	2020	9651	Cohort	Age, Sex	Fair
F Caramelo, et al (50)	China	2020	-	Descriptive exploratory analysis	Sex, Hypertension Diabetes mellitus, cardiac disease Respiratory failure, Malignancy	Fair
Kun Wang, et al (51)	China	2020	305	Cohort study	Age, Fever, Hypertension	Good
Qiao Shi, et al (24)	China	2020	79	Case control study	Diabetes mellitus	Good
Mingli Yuan, et al (52)	China	2020	27	Retrospective study	Sex, Hypertension, Diabetes mellitus cardiac disease, Malignancy, Respiratory failure, Fever, Cough Myalgia, Dyspnea	Good
Xiaobo Yang, et al(53)	China	2020	52	Retrospective study	Sex, cardiac disease, Chronic pulmonary disease, Diabetes mellitus Malignancy, Smoking, Fever Cough, Respiratory failure Myalgia, Headache, Respiratory failure Acute kidney injury	Good

### Assessment of effective factors on death of COVID-19

Eight studies had investigated the effect of the age on Mortality of COVID-19. The result of meta-analysis showed that the older age significantly effects on Mortality (OR=1.21, 95% CI: 1.10–1.33) with a significant between-study heterogeneity (*P*=<0.001, I^2^ =91.4%). There was a significant association between gender and death due to COVID-19. The male gender significantly affects Mortality (OR=1.41, 95% CI: 1.04–1.89). The heterogeneity across the 11 included studies was significant (*P*=0.005, I^2^ =74.5%). According to the overall result of meta-analysis on 9 studies, diabetes (type2) comorbidity showed significant effect on Mortality (OR=2.42, 95% CI: 1.06–5.52). With a significant between-study heterogeneity (*P*=0.001, I^2^ = 90.1%).

The analysis of 8 studies reported the data on Hypertension showed a significant effect of Hypertension comorbidity on Mortality (OR=2.54, 95% CI: 1.21–5.32) with a significant between-study heterogeneity (*P*=0.001, I^2^ =90.7%). According to the results of a meta-analysis of 7 records, reported data on kidney disorders comorbidity, having kidney disorders resulted in significant increase in odds of mortality with control group (OR=2.61, 95% CI: 1.22–5.60). The heterogeneity across the included studies was significant (*P*=0.001, I^2^=77.6%).

The results of a meta-analysis of 13 records had investigated the effect of the Respiratory disorders comorbidity on mortality, respiratory disorders significantly affect mortality (OR=3.09, 95% CI: 1.39–6.88). The heterogeneity across the included studies was significant (*P*= 0.001, I^2^ =81.4%).

Six studies had investigated the effect of the Heart disease comorbidity on mortality. The result of the meta-analysis showed that heart diseases significantly affect Mortality (OR=4.37, 95% CI: 1.13–16.90). The heterogeneity across the included studies was significant (*P*=0.001, I^2^ =88%).

Five studies had investigated the effect of the malignancy on mortality of COVID-19. Malignancy does not significantly affect mortality (OR=2.26, 95% CI: 0.73–6.22). The heterogeneity across the included studies was significant (*P*= 0.04, I^2^ =60.2%).

In three studies, Smoking resulted in increased risk of Mortality, but the observed effect was not significant (OR=1.7, 95% CI: 0.53–5.35). There was no significant heterogeneity among studies (I^2^ = 43.3%, *P*=0.17).

In total, 4, 6, 2 and 3 studies had investigated the effect of cough, fever, fatigue and headache, respectively on mortality of COVID-19. Clinical symptoms do not significantly affect mortality (for cough OR=0.78, 95% CI: 0.46–1.31, for fever OR=0.76, 95% CI: 0.35–1.68, for fatigue OR=1.52, 95% CI: 0.52–2.87, for headache OR=0.41, 95% CI: 0. 0.11–1.50). Regarding Clinical symptoms the heterogeneity across the included studies was not significant (for cough *P*=0.59, I^2^ =0.00%; for fever *P*=0.05, I^2^=53.4%; for fatigue *P*=0.72, I^2^=0.00%; for Headache *P*=0.57, I^2^=0.00%) (Table 2).

**Table 2:** Meta-analysis of Effective factors on Death of COVID-19 (random-effects model)

***Variables***	***Measure of association tools***	***Number of studies or records***	***Estimation***	***Q***	***I^2^***	***T^2^***
Age(yr)	Hazard Ratio(95% CI)	4	3.38(1.31–8.74)	<0.001	95.7	0.85
	Odds Ratio(95% CI)	4	1.13(1.08–1.18)	0.023	68.4	0.001
	Pooled	8	1.21(1.10–1.33)	<0.001	91.4	0.010
Sex	Hazard Ratio(95% CI)	4	1.06(0.51–2.18)	0.003	78.4	0.38
	Odds Ratio(95% CI)	7	1.70(1.30–2.21)	0.25	60.0	00.03
	Pooled	11	1.41(1.04–1.89)	<0.005	74.5	0.11
Diabetes(type2)	Hazard Ratio(95% CI)	2	1.96(1.19–3.22)	0.36	0.00	0.00
	Odds Ratio(95% CI)	7	2.63(0.91–7.25)	<0.001	90.0	1.59
	Pooled	9	2.42(1.06–5.52)	<0.001	90.1	1.26
Hypertension	Hazard Ratio(95% CI)	2	1.70(1.08–2.66)	1.00	0.00	0.00
	Odds Ratio(95% CI)	6	2.97(1.22–7.23)	<0.001	89.5	0.95
	Pooled	8	2.54(1.21–5.32)	<0.001	90.7	0.91
Kidney disorder	Hazard Ratio(95% CI)	3	6.33(3.46–11.57)	0.13	49.9	0.14
	Odds Ratio(95% CI)	4	1.35(0.57–3.22)	0.07	52.5	0.49
	Pooled	7	2.61(1.22–5.60)	<0.001	77.6	0.87
Malignancy	Odds Ratio(95% CI)	5	2.26(0.73–6.22)	0.04	60.2	0.83
Cough	Odds Ratio(95% CI)	4	0.78(0.46–1.31)	0.59	0.00	0.00
Fever	Hazard Ratio(95% CI)	1	0.41(0.20–0.81)	-	-	0.00
	Odds Ratio(95% CI)	5	0.97(0.38–2.48)	0.12	44.9	0.48
	Pooled	6	0.76(0.35–1.68)	0.05	53.4	0.46
Fatigue	Odds Ratio(95% CI)	2	1.52(0.52–2.87)	0.72	0.00	0.00
Headache	Odds Ratio(95% CI)	3	0.41(0.11–1.50)	0.57	0.00	0.00
Respiratory disorder	Odds Ratio(95% CI)	13	3.09(1.39–6.88)	<0.001	81.4	1.40
Heart diseases	Odds Ratio(95% CI)	6	4.37(1.13–16.90)	<0.001	88.0	2.15
Smoking	Odds Ratio(95% CI)	3	1.70(0.53–5.35)	0. 17	43.3	0.44

### Publication bias

There was no evidence of publication bias among the included articles assessing the effect factors on death of COVID-19 using Begg’s test (Age; *P*=1.0, Gender; *P*=0.2, Diabetes=0.3, Hypertension; *P*=0.7, Kidney disorders’=0.1, Malignancy=0.1, Cough; *P*=0.1, Fever; *P*=0.1, Fatigue; *P*=0.1, Headache; *P*=0.001, Respiratory disorder; *P*=0.4, Heart diseases; *P*=0.8, Smoking; *P*=0.1) and Egger’s tests (Age; *P*=1, Gender; *P*=0.1, Diabetes=0.03, HTN; *P*=0.06, Kidney disorders; *P*=0.1, Malignancy; *P*=0.001, Cough; *P*=0.7, Fever; *P*=0.1, Fatigue; *P*=0.1, Headache; *P*=0.02, Respiratory disorder; *P*=0.2, Heart diseases; *P*=0.1, Smoking; *P*=0.001).

### Meta-regression analysis

The effect of sample-size in different studies on the pooled measures was assessed. The sample size variation in different studies does not have a significant effect on heterogeneity between studies about different factors (*P*>0.05).

## Discussion

The COVID-19 is a highly contagious emerging disease that threatens people of all countries in the world (9). The disease has a wide range of clinical manifestations, from asymptomatic infection to severe and fatal pneumonia (10). Unfortunately, and only supportive therapies are available. On the other hand, the number of centers that provide intensive medical care as well as mechanical ventilator devices in many parts of the world is insufficient (4).

To date, more than 1,589,372 cases have been reported worldwide and from different countries (11). To deal with such an emerging infectious disease, there is an urgent need to identify and determine factors associated with evolution of the disease and its outcomes. In this systematic review and meta-analysis study, we reported the factors associated with death in patients with COVID-19.

The present study indicated the significant effects of age, gender and comorbidities such as diabetes mellitus, hypertension, kidney disorders and heart diseases on risk of death in patients with COVID-19.

The pooled analysis revealed older age is associated with higher risk of mortality may be due to lower levels of immune response at an older age. Older age is associated with declined immune competence (12). Older age is correlated with mortality in SARS (13) and MERS (14). Defects in T-cell and B-cell function and excess production of type 2 cytokines due to ageing could lead to an increase in viral replication and therefore to poor outcomes (15).

The present study showed that male gender significantly increases mortality (OR=1.41, 95% CI: 1.04–1.89. In a study on more than 44,000 Chinese cases, a CFR of 1.7% for females versus 2.8% for males has been reported (16). One study on 1099 patients with COVID-19 from 552 hospitals in China reported that only 42% of the patients were female (17). The fact that men are more susceptible to infection may be due to the X chromosome and sex hormones, reported to play role in innate and adaptive immunity (18). The effect of comorbidities on the prognosis of COVID-19 is similar to that reported for other respiratory infectious diseases such as MERS (19). Hypertension, diabetes, cardiovascular diseases, and respiratory system disease were amongst the most common comorbidities in COVID-19 patients (20). The diseases such as hypertension, diabetes, respiratory system diseases, cardiovascular diseases, could be linked to the pathogenesis of COVID-19 (20).

Our result showed that the diabetes mellitus (type 2) comorbidity had a significant effect on mortality (OR=2.42, 95% CI: 1.06–5.52). These findings are consistent with the concept that has been widely agreed upon in other studies that diabetic patients are at higher risk of infection, intensive Care Unit (ICU) admission and infection-related mortality (21–23). The mortality of diabetic patients with COVID-19 has been reported to be significantly higher and patients with diabetes are more prone to complications and secondary infections (24). The risk of hospitalization for diabetics following H1N1 flu has also been reported to be three times higher than in healthy individuals (25).

In diabetes, the accumulation of activated innate immune cells leads to the release of inflammatory mediators, especially, IL-1β and TNFα, which stimulates insulin resistance and β-cell damage (26). Diabetes Mellitus could lead to low immune function by macrophage and lymphocyte malfunctioning (27). This could predispose diabetic patients infected with COVID-19 to infections and severe complications or death (19). Moreover, levels of various inflammatory factors in the dead patients were significantly higher than those in survivors (28). Results of a study showed a significant reduction in CD3+ and CD8+ cell count in COVID-19 infected diabetic patients compared with non-diabetic individuals (29). Poor glycemic control in diabetic patients could impair immunity due to a decrease in T cell counts. The results of our study in line with previous studies showed that hypertension increases the risk of mortality in COVID-19 patients.

In China, hypertensive patients reported a more severe form of COVID-19 disease (20). Mortality among patients with MERS who had comorbidities such as diabetes and hypertension was more compared to patients who had no comorbidity (19).

Former studies (6, 30) on patients with kidney disorders have shown that they are more susceptible to infection with SARS and COVID-19 diseases. Acute Renal Impairment is a negative prognostic factor for survival in SARS patients (30). Chronic Kidney Disease (CKD) was associated with an increased risk of severe COVID-19 infection (6).

The results of the present study showed that having a respiratory disease significantly increases the risk of death in COVID-19 patients. Respiratory system diseases have been reported as a major risk factor for severe COVID-19 among hospitalized patients (20, 31).

The result of meta-analysis showed that heart diseases significantly affect mortality (OR=4.21, 95% CI: 1.19–14.82). Coronary heart disease has also been found to be associated with poor outcomes in influenza. Cardiovascular diseases have also been reported as a risk factor for MERS (32). A study on Animal has shown that viral RNA can be found in heart tissue, which can indicate viral RNA damages to the heart (33). A study reported a higher risk of death risk in influenza patients who had pulmonary disease, cardiovascular disease and hypertension (34).

Similar to our results a meta-analysis based on Chinese studies suggests that active smoking does not significantly increase the risk of severe disease in COVID-19 (35). In smokers level of angiotensin-converting enzyme 2 (ACE2) is lower than for non-smokers. ACE2 is the host receptor of the COVID-19 virus (36, 37). A non-significant association was reported between smoking and severity of COVID-19 (38).

The result of the current meta-analysis showed that malignancy does not significantly affect mortality (OR=2.26, 95% CI: 0.73–6.22). Patients with cancer are often immunosuppressed and are at the risk of severe complications (39). The CFR of COVID-19 disease among patients with cancer has been estimated to be 5.6% (40), and one study reported patients with cancer had higher risk of severe COVID-19 disease (41).

The result of the current meta-analysis showed that clinical symptoms do not significantly affect mortality. Fever is the most common symptom in patients with COVID-19, but not all patients had fever (42, 43).

This review has limitations considered when interpreting the results. First, a few studies are available for inclusion. Second, most studies were from China. Third, even though the current study was performed without any language restrictions and based on a comprehensive search strategy, only English electronic databases were searched; thus, some relevant non-English articles may have been missed.

## Conclusion

Infection with COVID-19 is associated with substantial mortality mainly in older patients with comorbidities. We found a significant effect of age, gender and comorbidities such as diabetes mellitus, hypertension, kidney disorders and heart diseases on risk of death among patients with COVID-19. The factors associated with mortality found in this research can help recognizing patients with COVID-19 who are at higher risk of a poor prognosis. Monitoring these factors can serve to give early warning for the appropriate interventions. To comprehensive understanding of the disease, data from countries that are currently facing a high prevalence of disease and mortality are needed. Additional research is also needed to reveal lesser-known aspects of the pathogenesis of severe and fatal infections.

## Ethical considerations

Ethical issues (Including plagiarism, informed consent, misconduct, data fabrication and/or falsification, double publication and/or submission, redundancy, etc.) have been completely observed by the authors.
